# The impact of fear of falling and social support on self-awareness of falls in older adult patients with cardiovascular multimorbidity: a multiple mediation model

**DOI:** 10.3389/fpubh.2026.1844615

**Published:** 2026-07-08

**Authors:** Ting Yao, Qiao Hu, Xiao Wang, Xiang Wang, Zheyuan Xia

**Affiliations:** 1 The School of Nursing, Anhui University of Chinese Medicine, Hefei, China; 2 Laboratory of Geriatric Nursing and Health, Anhui University of Chinese Medicine, Hefei, China

**Keywords:** chronic multimorbidity, fear of falling, path analysis, self-awareness of falls, social support

## Abstract

**Objective:**

To construct and validate a multiple mediation model of self-awareness of falls in older adult patients with cardiovascular multi-morbidity (CMM), and to elucidate the direct effects of fear of falling and social support on self-awareness of falls, as well as the mediating roles of emotion and cognitive function in these associations.

**Methods:**

A cross-sectional study was conducted. From September 2025 to February 2026, 358 older adult patients with cardio-metabolic multi-morbidity were recruited from 8 communities in Anhui Province by convenience sampling. Data were collected using a general information questionnaire, the Self-Awareness of Falls Scale (SAFE), the Falls Efficacy Scale-International (FES-I), the Hospital Anxiety and Depression Scale (HADS), the Perceived Social Support Scale (PSSS), and the Montreal Cognitive Assessment (MoCA). Univariate analysis, Pearson correlation analysis, and multiple linear regression analysis were performed using SPSS 26.0. Structural equation modeling and path analysis were conducted with AMOS 26.0, and the bias-corrected Bootstrap method was applied to examine the mediating effects.

**Results:**

The total score of self-awareness of falls in older adult patients with CMM was 61.27 ± 9.28. The structural equation model showed excellent fit (*χ*^2^/d*f* = 1.517, CFI = 0.962, TLI = 0.974, RMSEA = 0.038) and accounted for 62.9% of the variance in self-awareness of falls. Both fear of falling (*β* = 0.411) and social support (*β* = 0.356) directly and positively predicted self-awareness of falls. Mediation analysis indicated that anxiety and cognitive function served as significant parallel mediators between the two independent variables and self-awareness of falls. (1) Pathway “fear of falling → anxiety → self-awareness of falls” (indirect effect = 0.043, 95% CI: 0.009–0.087): fear of falling was positively correlated with anxiety (*r* = 0.187), suggesting that fall-related concern is associated with moderate risk anxiety, which in turn is associated with higher self-awareness of falls. (2) Pathway “fear of falling → cognitive function → self-awareness of falls” (indirect effect = 0.055, 95% CI: 0.025–0.102): fear of falling was significantly positively correlated with cognitive function (*r* = 0.252), suggesting that individuals with better cognitive function have a more accurate perception of their own fall risk. (3) Pathway “social support → anxiety → self-awareness of falls” (indirect effect = 0.083, 95% CI: 0.040–0.144): social support was significantly negatively correlated with anxiety (*r* = 0.287), and anxiety was positively correlated with self-awareness of falls (*r* = 0.490, *p* < 0.01), suggesting that social support may be associated with self-awareness of falls through a risk perception activation pathway, mediated positively by anxiety. (4) Pathway “social support → cognitive function → self-awareness of falls” (indirect effect = 0.068, 95% CI: 0.025–0.134): social support was significantly positively correlated with cognitive function (*r* = 0.295), suggesting that social support may enhance individuals’ ability to identify fall risks by promoting cognitive reserve, thereby associating with self-awareness of falls.

**Conclusion:**

In older adult patients with CMM, self-awareness of falls is directly and positively associated with fear of falling and social support, with anxiety and cognitive function serving as mediator variables, respectively. Clinical interventions should target precise management of anxiety: while utilizing the alerting function of fear, efforts should be made to strengthen social support networks and cognitive maintenance, so as to synergistically improve patients’ fall awareness.

## 1 Introduction

Falls are the leading cause of injury, disability and mortality among older adults. Epidemiological data show that the incidence of falls among older adults is 20.7% in China, 27.5% in the United States, and exceeds 50% among those aged 80 years and older in the United Kingdom (1–4). Against the backdrop of rapid population aging, multi-morbidity further elevates fall risk. Cardiovascular multi-morbidity (CMM), defined as the co-occurrence of two or more cardiovascular metabolic diseases (e.g., hypertension, diabetes mellitus, coronary heart disease, stroke), is one of the most prevalent multi-morbidity patterns in older adults, with reported prevalence rates ranging from 27.7 to 42.9% across countries (4, 5). As these conditions are frequently accompanied by hemodynamic instability, impaired balance, and central nervous system side effects attributable to polypharmacy, older adults with CMM exhibit a significantly higher fall risk than their healthy peers or those with a single chronic condition (6).

A study by Xiong et al. (7) estimated that 49.6% (95% CI: 45.9–53.2%) of older adults worldwide experience fear of falling. However, fear of falling is essentially a negative emotional experience and cannot be equated with an objective and rational perception of fall risk. The World Guidelines for Falls Prevention recognize an individual’s accurate identification of fall risk as the “first line of defense” in fall prevention. This multidimensional construct, encompassing risk identification, attention allocation, and behavioral intention, is defined as fall alertness (8). For older adults with CMM, maintaining an appropriate level of fall alertness enables individuals to proactively avoid high-risk situations while engaging in necessary activities, thereby reducing fall risk (8).

Existing studies on fall alertness have the following limitations. First, most studies have focused on the general older population or patients with a single disease, lacking targeted investigations of the high-risk group of CMM. Second, studies predominantly adopt variable-centered approaches, which only reveal isolated associations between single factors and fall alertness, failing to elucidate the complex pathways among multiple factors (9). Third, the possible emotional (anxiety, depression) and cognitive mediating pathways linking fear of falling, social support, and fall alertness have not yet been empirically tested.

To address these limitations, this study focused on older adults with CMM and adopted the Cognitive-Psychological Model as its theoretical framework. This model posits that an individual’s risk perception and behavioral decisions are jointly influenced by internal psychological factors and external environmental resources (10). Based on this framework, we constructed a multiple mediation model in which fear of falling and social support are associated with fall alertness through emotion and cognitive function. On the one hand, fear of falling, as a core psychological factor (11), is essentially an individual’s anticipatory cognitive and emotional response to potential fall risks and consequences (12). It may be associated with fall alertness via a cognitive pathway (regulating the allocation of cognitive resources) or an emotional pathway (inducing anxiety and depression). On the other hand, social support, as a key external environmental resource (13, 14), may be associated with fall alertness via a cognitive pathway (providing cognitive stimulation and preserving cognitive function) and an emotional pathway (buffering negative emotional interference). In summary, fear of falling and social support may not only be directly associated with fall alertness, but also indirectly associated through emotional and cognitive pathways.

Path analysis, a structural equation modeling technique for testing hypothesized relationships among variables (15), can effectively integrate multidimensional factors and quantify direct and indirect associations between variables. Accordingly, this study used path analysis to empirically test the above multiple mediation model, aiming to reveal the direct and indirect pathways through which fear of falling and social support are associated with fall alertness via emotion (anxiety, depression) and cognitive function in older adults with CMM. The findings are expected to deepen the understanding of factors related to fall alertness in this population and provide evidence for promoting the transformation of fall prevention strategies from “universal intervention” to “precision prevention and control.

## 2 Subjects and methods

### 2.1 Study design and participants

Using a convenience sampling method, 393 older adult patients with CMM were enrolled from 8 communities in Anhui Province, China, between September 2025 and February 2026.

*Inclusion criteria*: ① Age ≥65 years; ② Patients with CMM: having at least two cardiovascular and metabolic diseases, including hypertension, diabetes mellitus, dyslipidemia, heart disease, and stroke (2, 16). Hypertension: systolic blood pressure ≥140 mmHg (1 mmHg = 0.133 kPa) and/or diastolic blood pressure ≥90 mmHg, or a confirmed diagnosis of hypertension, or antihypertensive medication use in the past 2 weeks (17); Diabetes mellitus: a confirmed diagnosis of diabetes, or glucose-lowering treatment (oral hypoglycemic agents or insulin injection) in the past 2 weeks (18); Dyslipidemia: a confirmed diagnosis of dyslipidemia, or lipid-lowering medication use in the past 2 weeks, or meeting at least one of the following: LDL-C ≥ 4.1 mmol/L, TG ≥ 2.3 mmol/L, TC ≥ 6.2 mmol/L, and/or HDL-C < 1.0 mmol/L (19); Stroke: a history of stroke and/or transient ischemic attack (TIA), or receiving related treatment (20); Heart disease: a history of myocardial infarction, coronary heart disease, angina pectoris, congestive heart failure, or other cardiac diseases, or receiving related treatment (2). ③ Disease duration ≥6 months; ④ Clear consciousness, vision and hearing sufficient for normal reading and communication, and Barthel Index (BI) score >60.

*Exclusion criteria*: ① Presence of vital organ failure or malignant tumor; ② Severe cognitive impairment or history of psychiatric illness; ③ Current enrollment in other interventional studies.

*Sample size estimation*: In accordance with sample size requirements for structural equation modeling, the recommended sample size is generally at least 10 times the number of observed variables or 10–20 times the number of free parameters (21, 22). The present model included 17 observed variables, indicating a required sample size of 170–340. Taking into account the expected valid questionnaire response rate (≥90%), a target sample size of 187–374 participants was planned.

### 2.2 Study procedure

The research team comprised five members responsible for study management, quality control, data collection, and statistical analysis, respectively. All members had prior experience in community epidemiological surveys and received unified training before the study, including questionnaire interpretation, interview techniques, and standardized data entry procedures. Only those who passed a simulated survey and assessment were allowed to conduct formal investigations.

Written informed consent was obtained from all participants. For participants unable to read independently, the informed consent form was read aloud by the investigator, and a fingerprint was used instead of a signature in the presence of an independent witness. Surveys were administered face-to-face in community health offices or participants’ homes, with each interview lasting 15–25 min. Questionnaires were mainly self-completed by participants; for those with difficulties, investigators provided neutral assistance.

Review and data entry were completed within 24 h after questionnaire collection, and unclear information was verified in a timely manner. Questionnaires with a completion time of less than 10 min or regular response patterns were excluded. To ensure data accuracy, two researchers independently entered the data separately.

### 2.3 Variables and instruments

In the selection and inclusion of influencing factors, this study was grounded in the Cognitive-Psychological Model as its theoretical framework. After discussion by the research team, a total of 17 variables were finally included in the model. Demographic and disease-related data were collected using a general information questionnaire. Variables such as self-awareness of falls, fear of falling, anxiety and depression, social support, and cognitive function were assessed using standardized scales with well-established reliability and validity worldwide.

#### 2.3.1 Demographic and clinical instrument

Based on a literature review, a general information questionnaire was developed. It included 5 demographic items: age (65–74 years, 75–84 years, ≥85 years), sex, educational level, marital status, and living status (living alone; living with spouse/children or in a nursing home). Five disease-related variables were assessed: number of comorbidities (2, ≥3), Age-Adjusted Charlson Comorbidity Index (Age-Adjusted CCI), disease duration (<5 years, ≥5 years), number of medications (0; 1-2; ≥3), and history of falls in the past year (yes/no).

#### 2.3.2 Self-Awareness of Falls Scale (SAFE)

The scale was developed by Shyu et al. (8) to assess fall prevention alertness among older adults. It contains 21 items covering four dimensions: Awareness of Activity Safety and Environment (8 items), Awareness of Physical Functions (6 items), Awareness of Medication (3 items), and Awareness of Cognitive Behavior (4 items). All items were scored on a 5-point Likert scale, with items 1, 4, 6, 8, and 15 reverse-scored. Total scores range from 21 to 105, and a score >54 indicates a high level of self-awareness of falls (23). In the present study, the Cronbach’s *α* coefficient of this scale was 0.82.

#### 2.3.3 Falls Efficacy Scale-International (FES-I)

The scale was developed by Hauer et al. (24) to assess fear of falling during daily activities. It consists of 16 items covering two core dimensions: Activities of Daily Living at Home (8 items) and Outdoor and Social Activities (8 items). Items were scored on a 4-point Likert scale from 1 (“not worried at all”) to 4 (“very worried”), with total scores ranging from 16 to 64. Higher scores reflect greater fear of falling and lower fall-related self-efficacy. In the present study, the Cronbach’s *α* coefficient of this scale was 0.94.

#### 2.3.4 Hospital Anxiety and Depression Scale (HADS)

The scale was developed by Zigmond and Snaith (25) to assess anxiety and depression. It comprises two subscales: anxiety and depression, each containing 7 items. Items were scored on a 4-point Likert scale ranging from 0 (“no stress”) to 3 (“high stress”), with a total score of 0–21 for each subscale. A score ≥ 8 suggests the presence of clinically significant anxiety or depression. Higher scores indicate more severe anxiety and depression. In the present study, the Cronbach’s *α* coefficient of this scale was 0.87.

#### 2.3.5 Perceived Social Support Scale (PSSS)

The scale was developed by Zimet et al. (26) to assess perceived social support among individuals. It contains 12 items covering three dimensions: family support (4 items), friend support (4 items), and significant other support (4 items). Items were scored on a 7-point Likert scale from 1 (“strongly disagree”) to 7 (“strongly agree”), with total scores ranging from 12 to 84. Scores were positively correlated with social support level: total scores of 12–36 represented low social support, 37–60 moderate social support, and 61–84 high social support. In the present study, the Cronbach’s *α* coefficient of this scale was 0.82.

#### 2.3.6 Montreal Cognitive Assessment (MoCA)

The scale was developed by Nasreddine et al. (27) in 2005 for the rapid screening of mild cognitive impairment (MCI). It assesses eight cognitive domains, including visuospatial/executive function, naming, memory, attention, language, abstraction, and orientation, with a total of 30 items and a maximum score of 30. The score was adjusted for educational level: 1 point was added to the raw score for participants with ≤12 years of education. Higher scores indicate better cognitive function, with a standard cutoff of 26; a score <26 suggests cognitive impairment. In the present study, the Cronbach’s *α* coefficient of this scale was 0.84.

### 2.4 Statistical analysis

Data were double-entered and cross-checked by two independent researchers. Statistical analyses were performed using SPSS 26.0. Harman’s single-factor test was conducted to evaluate common method bias. Univariate analyses were performed using independent-samples *t*-test or one-way ANOVA; correlations among variables were examined using Pearson’s correlation analysis; and multivariate analysis was conducted using multiple linear regression. Structural equation modeling was established using AMOS 26.0. Model fit was assessed using the Chi-square-to-degree-of-freedom ratio (*χ*^2^/d*f*), comparative fit index (CFI), Tucker-Lewis index (TLI), and root mean square error of approximation (RMSEA). The thresholds for acceptable model fit were *χ*^2^/d*f* < 3.00, CFI > 0.90, TLI > 0.90, and RMSEA < 0.08. The significance of mediating effects was tested using the bias-corrected Bootstrap method with 5,000 resamplings, and 95% confidence intervals (CIs) of indirect effects were computed. A mediating effect was regarded as significant if the 95% CI did not include zero. The significance level was set at *α* = 0.05 (two-sided), and *p* < 0.05 was considered statistically significant.

### 2.5 Ethical considerations

This study was conducted in accordance with the ethical principles of the Declaration of Helsinki. The research protocol was approved by the Ethics Committee of Anhui University of Chinese Medicine (Approval No.: AHUCM-HSS-2025008). Written informed consent was obtained from all participants after full explanation of the study purpose and procedures. Data were collected via anonymous questionnaires and used exclusively for statistical analysis, with participants’ privacy fully protected throughout the study.

## 3 Results

### 3.1 Demographic and clinical characteristics

A total of 393 questionnaires were distributed, with 358 valid responses included in the final analysis (valid response rate: 91.09%). Participants ranged in age from 65 to 88 years (mean ± SD: 74.95 ± 6.94 years). Of the 358 participants, 183 (51.1%) were female and 175 (48.9%) were male; 230 (64.2%) had 2 cardiovascular conditions, and 128 (35.8%) had 3 or more; 68 (19.0%) reported a history of falls in the past year. Detailed demographic and clinical characteristics are presented in Table 1.

**Table 1:** Comparison of self-awareness of falls scores among older adult patients with CCM by sociodemographic and disease-related characteristics (*n* = 358).

Variables		Number of participants [*n* (%)]	Self-awareness of falls scores (分, x ± s)	*F*/*X*^2^	*p*-value
Gender	Female	183 (51.1%)	63.21 ± 10.02	4.148	<0.001^a^
	Male	175 (48.9%)	59.23 ± 7.96		
Age (year)	65~74	160 (44.7%)	58.57 ± 9.98	13.170	<0.001^b^
	75~84	164 (45.8%)	63.33 ± 7.21		
	≥85	34 (9.5%)	64.03 ± 11.39		
Education status	Senior high school and below	202 (56.4%)	61.73 ± 9.71	1.067	0.287^a^
	Bachelor’s degree and above	156 (43.6%)	60.67 ± 8.68		
Marital status	Married	268 (74.9%)	61.80 ± 8.80	1.873	0.062^a^
	Single or others	90 (25.1%)	59.69 ± 10.46		
Living situation	Living alone	114 (31.8%)	62.93 ± 10.36	2.331	0.020^a^
	Living with spouse/children or in a nursing home	244 (68.2%)	60.49 ± 8.64		
Number of comorbidities	2	230 (64.2%)	61.44 ± 9.31	0.467	0.641^a^
	≥3	128 (35.8%)	60.96 ± 9.24		
Disease duration (year)	<5	182 (50.8%)	60.76 ± 9.21	−1.046	0.296^a^
	≥5	176 (49.2%)	61.79 ± 9.34		
Number of regular medications	0	46 (12.8%)	58.17 ± 10.16	3.166	0.043^b^
	1-2	196 (54.7%)	61.47 ± 8.66		
	≥3	116 (32.4%)	62.15 ± 9.73		
History of falls within the previous 12 months	Yes	68 (19.0%)	64.24 ± 10.14	2.962	0.003^a^
	No	290 (81.0%)	60.57 ± 8.94		

a *t*.

b *F*.

### 3.2 Common method bias test

As self-reported scales were adopted in this study, Harman’s single-factor test was performed to evaluate potential common method bias and improve the rigor of the analysis. The results revealed 19 factors with eigenvalues greater than 1, and the first factor accounted for 22.52% of the total variance, which was below the 40% critical threshold. Accordingly, no severe common method bias was detected in the present data.

### 3.3 Scores of self-awareness of falls, fear of falling, anxiety, depression, social support and cognitive function in older adult patients with CMM

The total score on the Self-Awareness of Falls Scale (SAFE) was 61.27 ± 9.28, with a range of 34 to 99. Mean item scores for each dimension were: Awareness of Activity Safety and Environment (3.49 ± 0.66), Awareness of Physical Functions (3.48 ± 0.65), Awareness of Medication (2.43 ± 0.47), and Awareness of Cognitive Behavior (1.28 ± 0.36).

The total score on the Falls Efficacy Scale-International (FES-I) was 45.03 ± 6.74, ranging from 25 to 65. Mean item scores were: Activities of Daily Living at Home (2.97 ± 0.57) and Outdoor and Social Activities (2.66 ± 0.42).

For the Hospital Anxiety and Depression Scale (HADS), the total anxiety score was 7.41 ± 4.60 (range 0–18), with a mean item score of 1.06 ± 0.66; the total depression score was 7.05 ± 4.16 (range 0–17), with a mean item score of 1.01 ± 0.59.

The total score on the Perceived Social Support Scale (PSSS) was 45.35 ± 8.28, ranging from 19 to 75. Mean item scores for each dimension were: family support (4.52 ± 1.09), friend support (3.67 ± 0.84), and other support (3.16 ± 0.76).

The total score on the Montreal Cognitive Assessment (MoCA) was 22.96 ± 4.05, with a range of 15 to 28.

### 3.4 Univariate analysis of self-awareness of falls in older adult patients with cardiovascular multimorbidity

Differences in self-awareness of falls scores among older adult patients with CMM were statistically significant according to sex, age, living status, number of medications, and history of falls (*p* < 0.05), as detailed in Table 1.

### 3.5 Correlation analysis between self-awareness of falls and related variables in older adult patients with cardiovascular multimorbidity

The results of Pearson correlation analysis showed that self-awareness of falls in older adult patients with CMM was significantly positively correlated with the Age-Adjusted CCI, fear of falling, social support, anxiety, depression, and cognitive function, as shown in Table 2.

**Table 2:** Correlation analysis between study variables and self-awareness of falls (*r* values, *N* = 358).

Variables	Self-awareness of falls	Age-adjusted CCI	Fear of falling	Social support	Anxiety	Depression	Cognitive function
Self-awareness of falls	1						
Age-adjusted CCI	0.138**	1					
Fear of falling	0.425**	−0.008	1				
Social support	0.438**	0.044	0.156**	1			
Anxiety	0.490**	−0.008	0.187**	0.287**	1		
Depression	0.406**	0.032	0.333**	0.294**	0.137**	1	
Cognitive function	0.498**	0.036	0.252**	0.295**	0.297**	0.257**	1

*p < 0.05; **p < 0.01.

### 3.6 Multiple linear regression analysis of self-awareness of falls in older adult patients with cardiovascular multimorbidity

With self-awareness of falls as the dependent variable, variables showing statistical significance in univariate and correlation analyses were included as independent variables in multiple linear regression analysis (*α* entry = 0.05, *α* removal = 0.10). Dummy variables were created for the categorical variable “age” using the *k* − 1 principle, with 65–74 years as the reference category; for number of medications, 0 medications served as the reference. Males were the reference for sex; those living with spouse/children or in a nursing home were the reference for living status; and no history of falls was the reference for fall history. Continuous variables were entered as original values. All variables were entered using the forced-entry method.

Results indicated that age dummy variable 1 (75–84 years), sex, living status, history of falls, age-adjusted Charlson Comorbidity Index, fear of falling, anxiety, depression, social support, and cognitive function were all independent predictors of total self-awareness of falls scores in older adult patients with CMM (all *p* < 0.01). The overall model accounted for 62.9% of the total variance in self-awareness of falls (see Table 3).

**Table 3:** Results of multiple linear regression analysis of factors influencing self-awareness of falls in older adult patients with cardiovascular multimorbidity.

Variable	*b*	SE	*β*	*t*	*p*	Collinearity statistics	
						Tolerance	Variance inflation factor
Constant	20.205	3.856		5.240	<0.001		
Age (year)							
75~84	4.747	0.655	0.255	7.252	<0.001	0.874	1.144
≥85	2.379	1.252	0.075	1.900	0.058	0.686	1.457
Gender	−1.784	0.641	−0.096	−2.783	0.006	0.906	1.104
Living situation	−1.757	0.660	−0.088	−2.662	0.008	0.985	1.015
Number of regular medications							
1-2	1.425	0.965	0.077	1.476	0.141	0.403	2.483
≥3	1.928	1.030	0.097	1.871	0.062	0.401	2.497
History of falls within the previous 12 months	−2.145	0.793	−0.091	−2.705	0.007	0.967	1.034
Age-adjusted CCI	0.883	0.310	0.109	2.852	0.005	0.741	1.349
Fear of falling	0.355	0.050	0.258	7.162	<0.001	0.834	1.199
Anxiety	0.564	0.073	0.280	7.735	<0.001	0.829	1.207
Depression	0.358	0.084	0.161	4.288	<0.001	0.769	1.301
Social support	0.180	0.041	0.161	4.355	<0.001	0.793	1.261
Cognitive function	0.553	0.084	0.239	6.547	<0.001	0.816	1.225

*R* = 0.793, *R*^2^ = 0.629, adjusted *R*^2^ = 0.615, standard error of the estimate = 5.75351, *F* = 44.693, Durbin-Watson statistic = 2.158, *p* < 0.001.

### 3.7 Path analysis results of factors influencing self-awareness of falls in older adult patients with CMM

An initial structural equation model was established using AMOS 26.0, with self-awareness of falls (SAFE) as the dependent variable, fear of falling (FOF) and social support (PSSS) as independent variables, and anxiety (ANX), depression (DEP) and cognitive function (MoCA) as mediating variables.

Before model fitting using the maximum likelihood method, normality tests were conducted for each observed variable. The absolute values of skewness were all less than 2, and the absolute values of kurtosis were all less than 7, meeting the univariate normality criteria suggested by Kline (2016). Additionally, the Mardia coefficient was used to test multivariate normality, and the result was not significant (*p* > 0.05), satisfying the assumption of multivariate normality.

**Figure 1:**
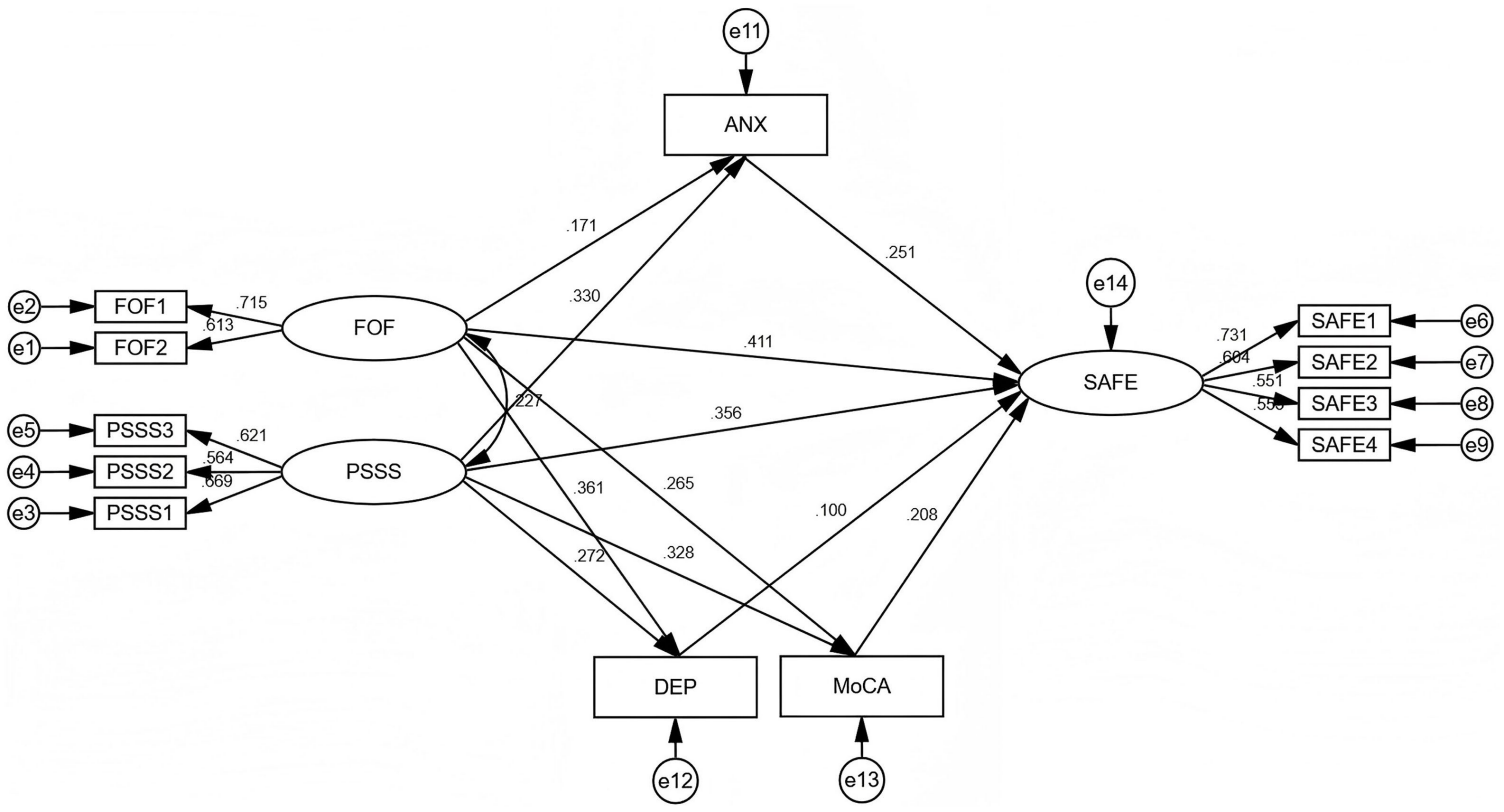
Structural equation model of influencing factors on fall alertness in older adult patients with CCM (Standardized).

The model was identifiable, and all variables were normally distributed. Model fitting was performed using the maximum likelihood estimation method. Fit indices were as follows: *χ*^2^ = 40.96, *χ*^2^/d*f* = 1.517, GFI = 0.969, AGFI = 0.947, RMSEA = 0.038, NFI = 0.930, RFI = 0.92, IFI = 0.975, TLI = 0.974, CFI = 0.962. The path diagram of the model is presented in Figure 1.

The bias-corrected nonparametric percentile Bootstrap method with 5,000 resamples was applied to test the mediating effects of fear of falling (FOF). Results were as follows: ① The total effect of FOF on self-awareness of falls (SAFE) was 0.546 (95% CI: 0.375–0.690, *p* < 0.001), including a direct effect of 0.411 (95% CI: 0.264–0.569, *p* = 0.001), accounting for 75.3% of the total effect, and a total indirect effect of 0.134 (95% CI: 0.062–0.232, *p* = 0.001), accounting for 24.7% of the total effect. ② Significant specific mediating pathways were: FOF → anxiety (ANX) → SAFE (indirect effect 1 = 0.043, 95% CI: 0.009–0.087, *p* = 0.018); FOF → cognitive function (MoCA) → SAFE (indirect effect 3 = 0.055, 95% CI: 0.025–0.102, *p* = 0.003). However, the pathway “FOF → depression (DEP) → SAFE” showed no significant mediating effect (indirect effect 2 = 0.036, 95% CI: −0.006 to 0.090, *p* = 0.081), as detailed in Table 4.

**Table 4:** Bootstrap mediation effect test of fear of falling (normalized).

Effect	Effect value	SE	95% CI^a^	*p*
Total effect	0.546	0.080	0.375~0.69	0.000
Direct effect	0.411	0.078	0.264~0.569	0.001
Total indirect effect	0.134	0.043	0.062~0.232	0.001
Indirect effect 1: FOF → ANX → SAFE	0.043	0.020	0.009~0.087	0.018
Indirect effect 2: FOF → DEP → SAFE	0.036	0.024	−0.006~0.090	0.081
Indirect effect 3: FOF → MoCA → SAFE	0.055	0.019	0.025~0.102	0.003

a The bias-corrected nonparametric percentile method was used.

Indirect effect 1: FOF → ANX → SAFE; Indirect effect 2: FOF → DEP → SAFE; Indirect effect 3: FOF → MoCA → SAFE.

The bias-corrected nonparametric percentile Bootstrap method with 5,000 resamples was used to examine the mediating effects of social support (PSSS). Results indicated that: ① The total effect of PSSS on self-awareness of falls (SAFE) was 0.535 (95% CI: 0.382–0.675, *p* < 0.001), including a direct effect of 0.356 (95% CI: 0.192–0.528, *p* = 0.001, 66.5% of the total effect) and a total indirect effect of 0.179 (95% CI: 0.091–0.286, p < 0.001, 33.5% of the total effect). ② Significant mediating pathways were: PSSS → anxiety (ANX) → SAFE (indirect effect 1 = 0.083, 95% CI: 0.040–0.144, *p* < 0.001); PSSS → cognitive function (MoCA) → SAFE (indirect effect 3 = 0.068, 95% CI: 0.025–0.134, *p* = 0.003). The pathway “PSSS → depression (DEP) → SAFE” showed no significant mediating effect (indirect effect 2 = 0.027, 95% CI: −0.005 to 0.069, *p* = 0.088), as presented in Table 5.

**Table 5:** Bootstrap mediation effect test of social support (standardized) (normalized).

Effect	Effect value	SE	95% CI^a^	*p*
Total effect	0.535	0.074	0.382~0.675	0.000
Direct effect	0.356	0.083	0.192~0.528	0.001
Total indirect effect	0.179	0.049	0.091~0.286	0.000
Indirect effect 1: PSSS → ANX → SAFE	0.083	0.025	0.040~0.144	0.000
Indirect effect 2: PSSS → DEP → SAFE	0.027	0.019	−0.005~0.069	0.088
Indirect effect 3: PSSS → MoCA → SAFE	0.068	0.027	0.025~0.134	0.003

a The bias-corrected nonparametric percentile method was used.

Indirect effect 1: PSSS → ANX → SAFE; Indirect effect 2: PSSS → DEP → SAFE; Indirect effect 3: PSSS → MoCA → SAFE.

It should be noted that this study employed a cross-sectional design, and the structural equation model reveals direct and indirect associative pathways among variables rather than causal mechanisms. All path coefficients should be interpreted as measures of the strength of association, not as causal relationships.

## 4 Discussion

### 4.1 Overall level and influencing factors of self-awareness of falls in older adult patients with CMM

In this study, the total score of self-awareness of falls in older adult patients with CMM was 61.27 ± 9.28, which was higher than that of community-dwelling older adults (52.94 ± 8.83) (28), and comparable to hospitalized older adult patients (62.67 ± 12.34) (29) and hospitalized older adult stroke patients (62.74 ± 11.52) (30). According to the cutoff criterion (SAFE > 54) (23), these results indicate that this population has developed a certain degree of vigilance against fall risks.

Univariate analysis revealed higher self-awareness of falls in female, older, living-alone, medication-exposed, and fall-experienced older adult patients with CMM. These findings may be attributed to fall-related negative experiences and increased exposure to fall hazards in daily life. They may also be related to fall risk perception, previous adverse fall events, insufficient daily care support, and physical discomfort and risk concerns caused by polypharmacy (31).

Multiple linear regression further identified independent predictors of self-awareness of falls, among which anxiety, fear of falling, and cognitive function were the three strongest core predictors (*β* = 0.280, 0.258, and 0.239, respectively). Age 75–84 years (*β* = 0.255), social support (*β* = 0.161), and sex (*β* = −0.096) also exerted significant effects. Notably, living status (*β* = −0.088) and comorbidity index (*β* = 0.109) remained significant in the regression model, suggesting that nurses should carry out individualized fall risk management by integrating objective disease burden and living environmental factors.

### 4.2 The association between fear of falling and self-awareness of falls and related indirect pathways

Fear of falling was a core determinant of self-awareness of falls in older adult patients with CMM, with a total effect of 0.546 and a direct effect of 0.411. Correlation analysis revealed a significant positive association between the two variables (*r* = 0.425, *p* < 0.01), suggesting that fear of falling can act directly as an adaptive warning signal and heighten individuals’ attention to fall risks. A U-shaped relationship has been reported between fear of falling and fall awareness (12): moderate fear activates the defensive vigilance system and improves awareness, whereas excessive fear impairs awareness by triggering generalized anxiety and depleting cognitive resources. In the present study, the mean score of fear of falling was 45.03 ± 6.74 (moderately high level), so only a positive linear effect was detected.

The two mediating pathways revealed indirect associations linking fear of falling to self-awareness of falls through emotional and cognitive routes. In the pathway “fear of falling → anxiety → self-awareness of falls” (indirect effect = 0.043, 95% CI: 0.009–0.087), fear of falling was positively associated with anxiety (*r* = 0.187). This pattern of associations indicates that concern about falling co-occurs with mild risk-related anxiety, which in turn co-occurs with higher levels of fall awareness.

In the pathway “fear of falling → cognitive function → self-awareness of falls” (indirect effect = 0.055, 95% CI: 0.025–0.102), fear of falling was significantly positively correlated with cognitive function (*r* = 0.252, *p* < 0.01). This result differs from previous reports of negative or no correlation (32). A plausible explanation for this positive correlation is that individuals with better cognitive function have a more accurate perception of their own fall risk and are more inclined to attend to fall prevention information and report moderate concern, rather than fear of falling itself improving cognitive function. In addition, selection bias (this study included hospitalized patients who were able to complete the MoCA and self-report scales, excluding those with severe cognitive impairment or extreme frailty; in a sample with relatively restricted cognitive function, the negative correlation may be weakened or reversed), residual confounding (e.g., physical function status, depressive mood), self-report bias, and conceptual overlap may all have contributed to the observed positive correlation.

It should be noted that anxiety was also significantly positively correlated with cognitive function in this study (*r* = 0.297, *p* < 0.01). The above explanations for the positive correlation between fear of falling and cognitive function also apply to this association. In summary, positive correlations between negative emotional variables and cognitive function in cross-sectional designs should be interpreted with caution. Furthermore, all correlations were based on self-report scales, and common method bias may have inflated their strength. Future studies should employ multi-source data to reduce bias.

### 4.3 The association between social support and self-awareness of falls and related indirect pathways

The total effect of social support on self-awareness of falls was 0.535, with a direct effect of 0.356 and an indirect effect of 0.134. The direct effect suggests that good social support directly enhances patients’ level of fall risk awareness. Care and assistance from family, friends, and the community not only provide concrete safety guarantees such as environmental modifications and medication reminders but also internalize fall prevention awareness into patients’ daily cognitive patterns through continuous social interaction.

Mediation analysis showed that anxiety exerted a significant mediating effect in the pathway from social support to self-awareness of falls (indirect effect = 0.083, 95% CI: 0.040–0.144). In this study, social support was positively correlated with anxiety (*r* = 0.287, *p* < 0.01), and anxiety was positively correlated with self-awareness of falls (*r* = 0.490, *p* < 0.01). These associations suggest that among older adults with CMM, higher levels of social support are associated with higher levels of anxiety, which in turn are associated with higher self-awareness of falls. This direction is opposite to the negative association predicted by stress-buffering theory (33). One possible explanation is that the social support received by patients is often accompanied by frequent health reminders and fall-related education. Although these behaviors help raise risk awareness, they may repeatedly reinforce patients’ perception of their own vulnerability, thereby being associated with higher anxiety levels. Therefore, in our sample, social support was associated with self-awareness of falls primarily through a risk-perception activation pathway (rather than an emotional buffering pathway) via the positive mediating role of anxiety. It should be noted that the cross-sectional design cannot rule out residual confounding or reverse causality, and the above mediating pathways should be interpreted as associations. Future longitudinal studies may employ fall-specific anxiety scales for further validation.

In the pathway “social support → cognitive function → self-awareness of falls” (indirect effect = 0.068, 95% CI: 0.025–0.134), social support was significantly positively correlated with cognitive function (*r* = 0.295, *p* < 0.01). Social interactions and daily activities maintained by social support provide continuous cognitive stimulation, which helps preserve executive function and attention related to fall risk perception (13), and thereby is associated with self-awareness of falls.

### 4.4 Comprehensive comparison and clinical implications

Comparison of the direct effects from the main pathways showed that fear of falling (*β* = 0.411) had a stronger predictive power for self-awareness of falls than social support (*β* = 0.356), suggesting that the independent effect of an individual’s own negative emotional experience on self-awareness of falls is more prominent than that of external social support resources. From the perspective of action pathways, fear of falling can directly activate risk vigilance through emotional arousal in a rapid manner. In contrast, the effect of social support on self-awareness of falls is population-specific. Rather than merely exerting a protective effect by buffering negative emotions and reducing anxiety, social support strengthens fall-related health education and attention to fall risk, thereby elevating individuals’ level of anxiety arousal and consequently enhancing fall awareness. This effect depends on the patient’s perception and internalization of externally provided support information. This also suggests that in clinical interventions, simply increasing social support to reduce negative emotions is insufficient; attention should also be paid to the risk-awareness-reinforcing effect of social support, and the optimal arousal range of anxiety should be explored and maintained.

In addition, depression was not significant in either mediating pathway. Although anxiety and depression were positively correlated (*r* = 0.137), the correlation between anxiety and self-awareness of falls (*r* = 0.490) was significantly stronger than that between depression and self-awareness of falls (*r* = 0.406). The reason is that anxiety, as an anticipatory emotion oriented toward future risk, is more closely linked to fall awareness—a prospective risk-related cognitive behavior—thereby masking the independent predictive effect of depression. Thus, anxiety plays a central role in the emotional regulation of fall awareness in older adults with CMM. Clinically, stratified screening and targeted management of anxiety should be prioritized over undifferentiated comprehensive interventions for all negative emotions.

In addition to the core predictors mentioned above, analysis of specific behavioral dimensions of self-awareness of falls showed that the mean item score for the cognitive-behavioral alertness dimension in older adult patients with CMM was 1.28 ± 0.36, which was significantly lower than that of other dimensions. Item content analysis indicated that low scores on this dimension reflect insufficient fall risk evaluation and weak willingness to seek help actively, which may be related to the traditional Chinese cultural value of “not wanting to trouble others.” Nurses should help patients correct this cognitive bias and use structured interventions such as cognitive restructuring and behavioral contracts (34) to enhance their willingness and behavior to seek care proactively.

Based on the above analyses and behavioral characteristics, clinical nursing practice can further focus on the following strategies. First, clinicians should abandon the stereotype that fear of falling is only a negative emotion, and integrate the adaptive warning value of moderate fear of falling into core fall risk screening for older adult patients with CMM. Individualized care plans can be formulated according to patients’ anxiety levels and cognitive function, converting moderate fear into proactive risk vigilance through emotion regulation and cognitive activation strategies, thus achieving integrated interventions for fall prevention and cognitive maintenance. Second, in light of the specific finding in this study that social support was positively associated with anxiety, conventional stress-buffering theory should not be applied mechanically with the sole goal of reducing anxiety. Instead, the optimal anxiety range that effectively triggers fall awareness should be explored, and social support networks should be used to reinforce positive risk education and moderately elicit risk concern, while avoiding excessive intervention that leads to over-anxiety. Furthermore, for high-risk groups with poor social support, insufficient risk awareness, and cognitive decline, intelligent monitoring technologies can be employed for dynamic fall risk assessment, and family and community support networks should be linked to deliver precise and individualized fall prevention management.

## 5 Limitations

This study has several limitations. First, the sample was recruited from eight communities in Anhui province using convenience sampling, which may have caused selection bias and somewhat limited the generalizability of the results to other regions. Second, the study adopted a cross-sectional design with all variables measured at one time point. Although this design can reveal covariation among variables, it cannot infer causal relationships or long-term dynamic changes among fear of falling, social support, and self-awareness of falls. Meanwhile, mediation tests based on cross-sectional data essentially reflect the strength of associations; longitudinal designs are needed in future research to verify the causal directions of the mediating pathways. Third, all variables were assessed using self-reported scales. Despite the good reliability and validity of the scales and acceptable common method bias, recall bias and social desirability may still exist, which could affect the objectivity of the results to some degree. Furthermore, there is some conceptual overlap among the scales used in this study (e.g., the SAFE, fear of falling, and anxiety scales all involve cognitive or emotional responses to fall risk), which may have inflated the correlation coefficients among the variables to some extent. Future studies should employ multi-source data (e.g., nursing assessments, objective cognitive tests) and longitudinal designs to reduce the impact of measurement overlap and method bias.

## 6 Conclusion

Based on the cognitive-psychological model, this study established and verified a multiple mediation pathway of self-awareness of falls in older adult patients with CMM. Results showed that fear of falling and social support were two core influencing factors, both of which directly and positively predicted self-awareness of falls. Further path analysis indicated that social support was associated with anxiety relief and preservation of cognitive function, which in turn were associated with higher self-awareness of falls. Fear of falling was associated with maintaining moderate anxiety and mobilizing cognitive resources, which in turn were associated with higher self-awareness of falls. This complementary pattern suggests that clinical interventions should take full advantage of the warning value of fear while strengthening social support networks, and keep fear at an appropriate level through emotional counseling and cognitive training, which in turn is associated with enhanced self-awareness of falls in patients.

## 7 Future directions

Future research can be extended in the following directions. First, multicenter prospective cohort studies should be performed to broaden sample sources and geographic coverage, track the dynamic changes in self-awareness of falls, and further verify the long-term effects and causal directions of fear of falling and social support. Second, based on the multiple mediating pathways identified in this study, intervention programs centered on “emotional regulation–cognitive training–social support enhancement” can be designed and verified via randomized controlled trials to evaluate their efficacy in improving fall awareness. Third, qualitative methods can be adopted to deeply explore patients’ psychological experiences and coping strategies in the face of fear of falling, so as to provide evidence for developing culturally sensitive and individualized fall prevention interventions.

## Data Availability

The raw data supporting the conclusions of this article will be made available by the authors, without undue reservation.
